# Correction: The determinant factors for the adoption of CRM in the Palestinian SMEs: The moderating effect of firm size

**DOI:** 10.1371/journal.pone.0259344

**Published:** 2021-10-26

**Authors:** Omar Hasan Salah, Zawiyah Mohammad Yusof, Hazura Mohamed

There is an error in affiliation 2 for authors Zawiyah Mohammad Yusof and Hazura Mohamed. The correct affiliation 2 is: Faculty of Information Science and Technology, Universiti Kebangsaan Malaysia, Selangor, Malaysia.
