# Supplementary material for: The determinant factors for the adoption of CRM in the Palestinian SMEs: The moderating effect of firm size
Source: PLoS One. 2021 Mar 4;16(3):e0243355. doi: 10.1371/journal.pone.0243355 (PMC7932763; doi:10.1371/journal.pone.0243355)
Supplement: S2 File — (DOCX) [file pone.0243355.s002.docx]

**QUESTIONNAIRE**

**Factors Affecting Customer Relationship Management System Adoption in Small and Medium Enterprise in Palestine**

Dear Sir / Madam,

Thank you for your cooperation for participation in completing this survey. I am a Ph.D. student in Management Information System at National University of Malaysia (UKM), we are working on a research titled “Factors Affecting Customer Relationship Management System Adoption in Small and Medium Enterprise in Palestine”. The main target of this survey is to identify the effect of critical success factor on CRM adoption in enhancing the SMEs performance in Palestine. Customer relationship management (CRM) is defined as a technology that manages the entire relationships and interactions between the organisation and its customers (present and potential) to improve business relationship. This questionnaire is purely for research purpose and your personal information will be kept private.

For further enquiry, please contact:

Name: Omar Salah

Email: omar_salah79@hotmail.com

Mobile: whatsApp Number 00972-597416617; mobile; 01162321482

CRM has become a key strategy for large and small companies alike, indicate the need for more SMEs to implement for effective business operations. CRM also develops a high-performance strategy and facilitate value-added, technical and innovative mechanisms to achieve the ultimate aim of obtaining a competitive edge over competitors (Mohamad et al. 2014).

Small and medium enterprises (SMEs) are referred to as such indicating their size, as economists tend to categorise businesses into classes based on quantitative measurable indicators. The top common criterion differentiating large and small businesses is the number of employed workers (Berisha & Pula 2015).with the macro enterprises employing less than 5 employees, small enterprise employing between (5-19), and medium enterprises employing (20-49) (Baidoun et al. 2018).

| **SECTION A: GENERAL INFORMATION** |
| --- |

This section is intended to obtain general information about you and your firm. Please put (√) to select the right choices

| 1. **Does your organization intend to adopt CRM technology?**  \|  \| \| --- \|   **Yes**   \|  \| \| --- \|   **No**   1. **Gender**  \|  \| \| --- \|   **Male**   \|  \| \| --- \|   **Female**   1. **Level of Education**  \|  \| \| --- \|   Diploma   \|  \| \| --- \|   Bachelor’s Degree   \|  \| \| --- \|   Master’s Degree   \|  \| \| --- \|   Doctorate Degree   1. **The type of your company**  \|  \| \| --- \|   [Information Communications Technology (ICT) BUSINESS](https://www.google.com/search?q=ICT+BUSINESS&spell=1&sa=X&ved=0ahUKEwi-h7HCk9rdAhUXU30KHZwcBvEQkeECCCgoAA)   \|  \| \| --- \|   Product companies  Service companies   \|  \| \| --- \|   Sales companies   \|  \| \| --- \| | 1. **You Position in organization.**  \|  \| \| --- \|   General Manager   \|  \| \| --- \|   Head of Department   \|  \| \| --- \|   Operational employees   1. **Number of employees in your organization**  \|  \| \| --- \|   Less than 5   \|  \| \| --- \|   5-19   \|  \| \| --- \|   20-49   \|  \| \| --- \|   More than 49   1. **Years of Experience**  \|  \| \| --- \|   <1 years   \|  \| \| --- \|   1-5 years   \|  \| \| --- \|   5-10 years   \|  \| \| --- \|   >10 years   1. **Age**  \|  \| \| --- \|   20-30 years   \|  \| \| --- \|   31-40 years   \|  \| \| --- \|   41-50 years   \|  \| \| --- \|   51 and above |
| --- | --- | --- | --- | --- | --- | --- | --- | --- | --- | --- | --- | --- | --- | --- | --- | --- | --- | --- | --- | --- | --- | --- | --- | --- | --- | --- | --- | --- |

| **SECTION (B): TECHNOLOGICAL FACTORS** |
| --- |

Please indicate the extent to which you are agree or disagree of the following statement regarding technology factors that affect CRM adoption in your company (1: Strongly Disagree; 2: Disagree; 3: Slightly agree; 4: Agree; 5: Strongly Agree):

| **Compatibility** | | | | | | |
| --- | --- | --- | --- | --- | --- | --- |
| No | Indicator | 1 | 2 | 3 | 4 | 5 |
| 1 | The adoption of CRM technology is consistent with all aspects of my company. |  |  |  |  |  |
| 2 | CRM technology is compatible with customers’ ways of doing business. |  |  |  |  |  |
| 3 | Transformation towards CRM technology adoption in our company is favorable. |  |  |  |  |  |
| 4 | CRM technology is compatible with our company’s current software. |  |  |  |  |  |
| 5 | CRM technology is compatible with our company’s current hardware. |  |  |  |  |  |
| **IT Infrastructure** | | | | | | |
| No | Indicator | 1 | 2 | 3 | 4 | 5 |
| 1 | Computers are provided for all employees. |  |  |  |  |  |
| 2 | My organization has a high degree of information system interconnectivity. |  |  |  |  |  |
| 3 | The data will be available to everyone in my organization by using CRM technology. |  |  |  |  |  |
| 4 | The adoption of CRM is important to share knowledge with my co-workers. |  |  |  |  |  |
| 5 | New locations or acquisitions are quickly assimilated into my IT infrastructure. |  |  |  |  |  |
| **Complexity** | | | | | | |
| No | Indicator | 1 | 2 | 3 | 4 | 5 |
| 1 | The skills required to use CRM technology are too complex for the employees in the company. |  |  |  |  |  |
| 2 | It is difficult for us to integrate the CRM technology to the current business operations. |  |  |  |  |  |
| 3 | Lack of appropriate tools to use CRM technology. |  |  |  |  |  |
| 4 | Using CRM technology needs a lot of mental effort. |  |  |  |  |  |
| 5 | Company lacks adequate computer systems to support CRM technology. |  |  |  |  |  |
| **Relative advantage** | | | | | | |
| No | Indicator | 1 | 2 | 3 | 4 | 5 |
| 1 | The adoption of CRM technology helps the company to increase customer base. |  |  |  |  |  |
| 2 | The adoption of CRM technology cuts costs in operation. |  |  |  |  |  |
| 3 | The adoption of CRM reduces order response time |  |  |  |  |  |
| 4 | The adoption of CRM helps generate competitive advantage. |  |  |  |  |  |
| 5 | The adoption of CRM technology provides timely and accurate information for decision making. |  |  |  |  |  |
| **Security** | | | | | | |
| No | Indicator | 1 | 2 | 3 | 4 | 5 |
| 1 | The organisation protects its information assets adequately by adopting CRM technology. |  |  |  |  |  |
| 2 | It is important to understand the threats to the information assets (for example, systems and information) in my department. |  |  |  |  |  |
| 3 | Threats to security of information assets (for example, CRM) are controlled adequately in my department. |  |  |  |  |  |
| 4 | I believe my business unit will survive when using CRM technology if there is a disaster resulting in the loss of systems, people and/or premises. |  |  |  |  |  |
| 5 | Since the CRM technology has adequate security features, I feel that my privacy is protected at this system. |  |  |  |  |  |
| 6 | Assurance of security as a factor is important in choosing CRM technology. |  |  |  |  |  |

| **SECTION (C): ORGANIZATIONAL FACTORS** |
| --- |

Please indicate the extent to which you are agree or disagree of the following statement regarding organizational factors that affect CRM adoption in your company (1: Strongly Disagree; 2: Disagree; 3: Slightly agree; 4: Agree; 5: Strongly Agree):

.

| **Top management support** | | | | | | |
| --- | --- | --- | --- | --- | --- | --- |
| No | Indicator | 1 | 2 | 3 | 4 | 5 |
| 1 | Relative importance is given by the top management to adopt CRM technology. |  |  |  |  |  |
| 2 | Our management have clear quality goals identified by top-level managers for adopting CRM technology. |  |  |  |  |  |
| 3 | The management is committed to adopting CRM technology. |  |  |  |  |  |
| 4 | The top management has a desire to adopt CRM technology to improve the competitive strategies of the company. |  |  |  |  |  |
| 5 | our management is very concerned with the performance of the CRM technology. |  |  |  |  |  |
| 6 | Top management makes an effort to provide sufficient funding for CRM adoption and operation. |  |  |  |  |  |
| **Employee Engagement** | | | | | | |
| No | Indicator | 1 | 2 | 3 | 4 | 5 |
| 1 | Teamwork is encouraged and practised in this organization. |  |  |  |  |  |
| 2 | There is a strong feeling of teamwork and cooperation in this organization. |  |  |  |  |  |
| 3 | This organization is extremely focused on customer needs. |  |  |  |  |  |
| 4 | In this organization we maintain very high standards of quality. |  |  |  |  |  |
| 5 | The quality of our products and services are very important to this organization. |  |  |  |  |  |
| 6 | This organization has high performance standards. |  |  |  |  |  |
| **Information policies** | | | | | | |
| No | Indicator | 1 | 2 | 3 | 4 | 5 |
| 1 | Information in my organization is easy to access at any time I need. |  |  |  |  |  |
| 2 | My organization has formal procedures or policies to collect information regarding best practices and current research. |  |  |  |  |  |
| 3 | My organization has a culture that promotes knowledge and information sharing among employees. |  |  |  |  |  |
| 4 | My organization makes use of information technology for information sharing. |  |  |  |  |  |
| 5 | The information policies of the organization are clearly stated. |  |  |  |  |  |
| 6 | The information policies of the organization are well known to every employee. |  |  |  |  |  |
| 7 | In this company, decisions are made at those levels where the most accurate information is available. |  |  |  |  |  |
| **Financial resource** | | | | | | |
| No | Indicator | 1 | 2 | 3 | 4 | 5 |
| 1 | The amount of money and time to be invested to adopt CRM technology are low. |  |  |  |  |  |
| 2 | The cost of CRM technology is quite low for my company. |  |  |  |  |  |
| 3 | Availability of internal financial resources is positively related to CRM technology in my companies. |  |  |  |  |  |
| 4 | The equipment required to use CRM technology is not expensive. |  |  |  |  |  |
| 5 | My company will take CRM technology more seriously when receiving adequate financial support. |  |  |  |  |  |

**SECTION (D): ENVIRONMENTAL FACTORS**

Please indicate the extent to which you are agree or disagree of the following statement regarding information culture factors that affect CRM adoption in your company (1: Strongly Disagree; 2: Disagree; 3: Slightly agree; 4: Agree; 5: Strongly Agree):

.

| **Customers Pressure** | | | | | | |
| --- | --- | --- | --- | --- | --- | --- |
| No | Indicator | 1 | 2 | 3 | 4 | 5 |
| 1 | Our customers are pressuring us to adopt CRM technology. |  |  |  |  |  |
| 2 | Customers’ requirements indicate that our company need to adopt CRM technology. |  |  |  |  |  |
| 3 | Customers’ behaviours indicate that our company need to adopt CRM technology. |  |  |  |  |  |
| **Competitive pressure** | | | | | | |
| No | Indicator | 1 | 2 | 3 | 4 | 5 |
| 1 | We believe that we will lose our customers to our competitors if we do not adopt CRM technology. |  |  |  |  |  |
| 2 | We feel there is a strategic necessity to use CRM technology to compete in the market. |  |  |  |  |  |
| 3 | Our organization has experienced competitive pressure to adopt CRM technology. |  |  |  |  |  |
| 4 | Our organization would experience a competitive disadvantage if CRM technology services is not adopted. |  |  |  |  |  |

| **SECTION (D): INFORMATION CULTURE FACTORS** |
| --- |

Please indicate the extent to which you are agree or disagree of the following statement regarding information culture factors that affect CRM adoption in your company (1: Strongly Disagree; 2: Disagree; 3: Slightly agree; 4: Agree; 5: Strongly Agree):

.

| **Attitude toward adoption technology in the organization** | | | | | | |
| --- | --- | --- | --- | --- | --- | --- |
| No | Indicator | 1 | 2 | 3 | 4 | 5 |
| 1 | CRM technology increases the productivity of my employees. |  |  |  |  |  |
| 2 | My employees find CRM technology is easy to use |  |  |  |  |  |
| 3 | 1 have seen what other small companies have achieved with CRM technology. |  |  |  |  |  |
| 4 | The adoption of CRM technology will be accepted by staff in my organization. |  |  |  |  |  |
| **Information sharing** | | | | | | |
| No | Indicator | 1 | 2 | 3 | 4 | 5 |
| 1 | Information sharing is encouraged within my organization. |  |  |  |  |  |
| 2 | My organization has a culture of sharing information. |  |  |  |  |  |
| 3 | My organization values information sharing. |  |  |  |  |  |
| 4 | Information sharing is practiced by employees. |  |  |  |  |  |
| 5 | we often exchange information with partner organizations. |  |  |  |  |  |
| **Information integrity** | | | | | | |
| No | Indicator | 1 | 2 | 3 | 4 | 5 |
| 1 | it is common to knowingly pass on accurate information within our work environment  in the organization. |  |  |  |  |  |
| 2 | Our people frequently distribute information to justify decisions after the fact |  |  |  |  |  |
| 3 | Our people frequently keep information to themselves |  |  |  |  |  |
| 4 | Employees frequently do not exploit business information for personal gain. |  |  |  |  |  |
| 5 | The strong personal integrity of employee enables effective sharing of sensitive |  |  |  |  |  |

| **SECTION (E): CRM Adoptions** |
| --- |

Please indicate the extent to which you are agree or disagree of the following statement regarding CRM adoption in your company (1: Strongly Disagree; 2: Disagree; 3: Slightly agree; 4: Agree; 5: Strongly Agree):

.

| **CRM Adoptions** | | | | | | |
| --- | --- | --- | --- | --- | --- | --- |
| No | Indicator | 1 | 2 | 3 | 4 | 5 |
| 1 | my organization have intention to adopt CRM technology in our operation in near future |  |  |  |  |  |
| 2 | CRM technology is important to my work and we need to adopt it |  |  |  |  |  |
| 3 | My organization has sought for adoption of CRM technology in our work. |  |  |  |  |  |
| 4 | We have well-planned to adopt the CRM in our work. |  |  |  |  |  |
| 5 | my organization considered the adoption of CRM in our work. |  |  |  |  |  |

| **SECTION (F): ORGANIZATIONAL PERFORMANCE** |
| --- |

Please indicate the extent to which you are agree or disagree of the following statement regarding CRM adoption in your company (1: Strongly Disagree; 2: Disagree; 3: Slightly agree; 4: Agree; 5: Strongly Agree):

| **Organizational performance** | | | | | | | |
| --- | --- | --- | --- | --- | --- | --- | --- |
| No | Indicator | 1 | 2 | 3 | 4 | 5 |  |
| 1 | My company has a greater market share after the adoption of CRM technology |  |  |  |  |  |  |
| 2 | Our profitability rate is higher than it was before the adoption of CRM technology |  |  |  |  |  |  |
| 3 | The adoption of CRM technology in my organization will increase the sales growth rate |  |  |  |  |  |  |
| 4 | The adoption of CRM technology will increase the relationship between my company and its customers |  |  |  |  |  |  |
| 5 | The relationship between my company and its customers reflect a happy situation after adoption of CRM technology |  |  |  |  |  |  |

**Thank You for Your Co-operation**
