# Supplementary material for: The determinant factors for the adoption of CRM in the Palestinian SMEs: The moderating effect of firm size
Source: PLoS One. 2021 Mar 4;16(3):e0243355. doi: 10.1371/journal.pone.0243355 (PMC7932763; doi:10.1371/journal.pone.0243355)
Supplement: Attachment — Submitted filename: PLOS ONE reviewer report PONE-D-19-33183.pdf [file pone.0243355.s003.pdf]

PLOS ONE reviewer report

PONE-D-19-33183

Paper: *The Determinant Factors for the Adoption of CRM in the Palestinian SMEs: The Moderating Effect of Firm Size*

Report

This is an interesting paper covering the topic of customer relationship management, specifically on the adoption of such practices in the case of SMEs in Palestine.

While the paper employs a reliable methodology for this sort of research and some interesting results are reported (which could also prove useful for certain stakeholders), there are still much to be improved from the paper.

Some of my comments (concerns) about the paper are as follows:

- Overall, there is still much to do to improve the writing especially on the grammar. The standard of writing appears to be a little inconsistent, with some parts fairly well-written but others littered with grammatical errors and editorial issues. It would be good to have this paper edited by a professional proofreader or at the very least have someone (whose first language is English) go through it.
- The introduction section (on business and the use of IT in its processes) should be a little longer and a little more general before the paper jumps into the *CRM* materials.
- A section (or sub-section) devoted to *CRM* after the introduction section would improve the flow of the paper and also at the same time, provide a sort of primer on the topic of *CRM* for the readers especially those not too familiar with the topic.
- Following the above point, there is also a need to restructure things a little bit on the flow of first few sections - I would suggest that the author(s) use some numbering on the sections and sub-sections ----- for e.g., *1.0 Introduction; 2.0 CRM; 2.1 Importance & Drawbacks of CRM; 2.2 Adoption of CRM; 2.3 Requirements for the Adoption of CRM*; etc..
- A section should also be devoted to an overview of the Palestine economy, market and SMEs in the country --- this should form a section, for e.g. Section *3.0 The Palestine Market and SMEs*
- As many parts in the section on "Palestine and the Requirement for the Adoption of CRM" are quite generic, those parts should be placed in section *2.3 Requirements for the Adoption of CRM* instead.
- A more detailed discussion should be directed at the Palestine economy, the state of the SMEs and the SMEs' current experience and readiness in embracing technology (including CRM) in their business processes should be

provided. At present, they are too skeletal and casual (located at different parts of the essay as well) to provide any sort of understanding and interest to the readers. This part should form section *3.0 The Palestine Market and SMEs*.

- A critical section devoted to the literature review on *CRM* and the experience of Palestine/Middle Eastern countries' SMEs with *CRM* is needed to better inform readers on the current development of the topic. If there are no papers on this, then the findings of this paper would be the key contribution of the paper and if so, this should have been stated clearly at the onset of the paper.
- In page 11 (line 5), the authors stated "It is pertinent for SMEs in Palestine to adopt *CRM* for ….…." – why Palestine? There is a need to provide some context especially since this is the country selected in the paper. It would be good if the authors state why they opted to study SMEs in Palestine (as opposed to other countries in the Middle East). This point relates to the issues of both (a) the motivation of the paper and (b) the problem statement. The author(s) may want to consider incorporating these (optional though) into the paper - this provides greater clarity and intention of the paper.
- I have no issues with the section *'Factors Influencing CRM Adoption'* but it would be better to provide a critical literature review of this (i.e., past papers on the factors 1-8) – the list of references are provided in Table 1 but a critical review of them would be preferred. That said, I noticed that the review is somewhat provided in the section "*Factors Influencing CRM adoption*' in page 11 but that section appears to be used instead as 'justifications for the moderating effects of size on those 8 factors', hence the subsequent hypotheses (8 of them). I am not persuaded that the authors have succeeded in linking size to the effects of those 8 factors have on *CRM* adoption.
- I have some issue though with the section "*Moderating effect of firm size on CRM adoption*" – the authors need to make clearer the contents in Table 2. I understand that it is about firm size and CRM adoption but it's a little convoluted, not clearly explained and also had parts that are only discussed after the section – e.g. 'relative advantages', 'compatibility', 'top management support' etc… These appear only in the subsequent section (i.e., *Theoretical Background* section). Also, certain parts (boxes) in Table 2 are left empty – why? The author(s) also used the term *CSF* in this section but did not state what it stands for. The paragraphs under this section should have done the job of explaining the moderating effects of firm size and also provide the literature review of it (i.e., the moderating effects of firm size on….)
- The section on '*Theoretical Background'* in page 13 should precede the section on '*Factors Influencing CRM Adoption*' as they provide some context

(e.g., the environment and development in which *CRM* adoption taking place) under which the question of CRM adoption is being addressed.

- I don't want to nit-pick but there seems to be some inconsistency/errors in referencing formats as well – the author(s) really do need to conduct better editorial work.
- Some alignment adjustments of the figures (in the row 'type of company') in Table 3 are needed.
- Insert a Figure 3 in page 35.
- In the sample profile section, the author(s) may want to consider providing more information as to the type of industry as well. For e.g., a sales company, product and service company tell little about the industry. I suspect that *CRM* may matter more for certain industries than others so such information may be useful here. The author(s) also mentioned this in the last sentence of the paper (last sentence in the '*conclusion*' section).
- Not sure if this matters (since I did not see the survey questions) but wouldn't it be better to target respondents who are more in a managerial capacity? I noticed that the paper included operational employees, presumably these are also younger and very junior level employees (e.g., there is a '20-30 years old category' in the sample)
- I'm not questioning the validity of the results but wouldn't it make sense (or at the very least make clearer) to test for significance of the 8 factors' effects on *CRM* adoption first before analyzing the effects of firm size on these factors' impact on *CRM* adoption. At the very least, the author(s) may want to make it clearer of the results and methods used in deriving the findings in Table 4.
- In the figures 4-7, the results of firm size are presented but they appeared to be based only on whether the firm is a small or medium size firm. E.g. for Fig. 7, size has a moderating effect on top management support (TMS) when it comes to *CRM* adoption in the case of medium size but not small size firm? How do the author(s) define the cutoff between small and medium? And what does the vertical axis in Figures 4-7 stand for?

Overall, I think the paper does have some interesting results but the author(s) can do a better job in presenting them in my opinion. There's a need to provide greater clarity in the methodology employed and how they are used to measure the intended effects on CRM adoption, i.e., the effects of size. I also think that they should make a case as to why SEM is chosen as opposed to other methodologies (e.g. regression, interviews etc..). For that, some discussions on SEM (PLS-SEM) should have been inserted into the section on '*methods*' in page 19. Also the '*methods*' section should be changed to '*Data & Methodology*'. As such, I suspect the paper may have been a part of a much larger piece of work (e.g. a dissertation/thesis?) thus the author(s) may want to do a tighter and better editorial effort to ensure a more coherent and informed piece of work.
