# Supplementary material for: The determinant factors for the adoption of CRM in the Palestinian SMEs: The moderating effect of firm size
Source: PLoS One. 2021 Mar 4;16(3):e0243355. doi: 10.1371/journal.pone.0243355 (PMC7932763; doi:10.1371/journal.pone.0243355)
Supplement: Attachment — Submitted filename: Comments.docx [file pone.0243355.s004.docx]

| Comments | page |
| --- | --- |
| The introduction section (on business and the use of IT in its processes) should be a little longer and a little more general before the paper jumps into the CRM materials | 1 |
| A section (or sub-section) devoted to CRM after the introduction section would improve the flow of the paper and also at the same time, provide a sort of primer on the topic of CRM for the readers especially those not too familiar with the topic. | 2+ 3+4 |
| A section should also be devoted to an overview of the Palestine economy, market and SMEs in the country --- this should form a section, for e.g. Section 3.0 The Palestine Market and SMEs | 5+6 |
| Following the above point, there is also a need to restructure things a little bit on the flow of first few sections - I would suggest that the author(s) use some numbering on the sections and sub-sections ----- for e.g., 1.0 Introduction; 2.0 CRM; 2.1 Importance & Drawbacks of CRM; 2.2 Adoption of CRM; 2.3 Requirements for the Adoption of CRM; etc | Done |
| As many parts in the section on “Palestine and the Requirement for the Adoption of CRM” are quite generic, those parts should be placed in section 2.3 Requirements for the Adoption of CRM instead. | Section 2.4 page 4 |
| A more detailed discussion should be directed at the Palestine economy, the state of the SMEs and the SMEs’ current experience and readiness in embracing technology (including CRM) in their business processes should be provided. At present, they are too skeletal and casual (located at different parts of the essay as well) to provide any sort of understanding and interest to the readers. **This part should form section 3.0 The Palestine Market and SMEs**. • | page 5 |
| In page 11 (line 5), the authors stated “It is pertinent for SMEs in Palestine to adopt CRM for” – why Palestine? There is a need to provide some context especially since this is the country selected in the paper. It would be good if the authors state why they opted to study SMEs in Palestine (as opposed to other countries in the Middle East). This point relates to the issues of both (a) the motivation of the paper and (b) the problem statement. The author(s) may want to consider incorporating these (optional though) into the paper - this provides greater clarity and intention of the paper. | Page 5 |
| I noticed that the review is somewhat provided in the section “Factors Influencing CRM adoption’ in page 11 but that section appears to be used instead as ‘**justifications for the moderating effects of size on those 8 factors’**, hence the subsequent hypotheses (8 of them) | Page 5 |
| e section – e.g. ‘relative advantages’, ‘compatibility’, ‘top management support’ etc… These appear only in the subsequent section (i.e., Theoretical Background section). Also, certain parts (boxes) in Table 2 are left empty – why? The author(s) also used the term CSF in this section but did not state what it stands for. The paragraphs under this section should have done the job of explaining the moderating effects of firm size and also provide the literature review of it (i.e., the moderating effects of firm size on….) | Done |
| In the sample profile section, the author(s) may want to consider providing more information as to the type of industry as well. For e.g., a sales company, product and service company tell little about the industry. I suspect that CRM may matter more for certain industries than others so such information may be useful here. The author(s) also mentioned this in the last sentence of the paper (last sentence in the ‘conclusion’ section). | Page 16 |
| Not sure if this matters (since I did not see the survey questions | I attached my questionnaire |
| At the very least, the author(s) may want to make it clearer of the results and methods used in deriving the findings in Table 4 | Page18 Findings and discussion |
| Define the cutoff between small and medium? And what does the vertical axis in Figures 4-7 stand for? | Page 16 |
