# Supplementary material for: The determinant factors for the adoption of CRM in the Palestinian SMEs: The moderating effect of firm size
Source: PLoS One. 2021 Mar 4;16(3):e0243355. doi: 10.1371/journal.pone.0243355 (PMC7932763; doi:10.1371/journal.pone.0243355)
Supplement: Attachment — Submitted filename: Comments.docx [file pone.0243355.s005.docx]

| Comments | Page |
| --- | --- |
| The structure of the article must have four very clear sections: introduction, method, results and discussion and conclusion. Authors should make any necessary adjustments to provide this clarity. In this regard, authors must place point 5. justifications for using PLS within the Method section. | 17 |
| - The name of the Ethics Committee that supervised the study must be stated. This aspect is resolved. The date of the study must appear in the Method section. | 17 |
| he methodology used (The Partial Least Square-Structural Equation Model (PLS-SEM)) is not justified, nor does it seem to be specified in the text or it is not made clearly.. | 17 |
